# Optimized T_1_ ‐weighted MP‐RAGE MRI of the brain at 0.55 T using variable flip angle coherent gradient echo imaging and deep learning reconstruction

**DOI:** 10.1002/mrm.70109

**Published:** 2025-09-29

**Authors:** Oliver Bieri, Marcel Dominik Nickel, Claudia Weidensteiner, Philipp Madörin, Grzegorz Bauman

**Affiliations:** ^1^ Department of Biomedical Engineering University of Basel Allschwil Switzerland; ^2^ Department of Radiology, Division of Radiological Physics University Hospital Basel Basel Switzerland; ^3^ Research & Clinical Translation, Magnetic Resonance Siemens Healthineers AG Erlangen Germany

**Keywords:** deep learning reconstruction, image enhancement, low‐field, MP‐RAGE, MRI, T_1_‐weighted brain

## Abstract

**Purpose:**

To propose and evaluate an optimized MP‐RAGE protocol for rapid T_1_‐weighted imaging of the brain at 0.55 T.

**Methods:**

Incoherent and coherent steady state free precession (SSFP) RAGE kernels with constant and variable excitation angles were investigated in terms of the white matter SNR and the white matter–gray matter signal difference. Potential edge smearing from the transient signal readout was assessed based on a differential point spread function analysis. Finally, the prospects of a deep‐learning reconstruction (DLR) method for accelerated MP‐RAGE MRI of undersampled data were evaluated for the best performing variant.

**Results:**

MP‐RAGE imaging with a variable flip angle (vFA) SSFP‐FID kernel outperformed all other investigated variants. As compared to the standard MPRAGE sequence using a spoiled gradient echo kernel with constant flip angle, vFA SSFP‐FID offered an average gain in the white matter SNR of 21% ± 2% and an average improvement for the white matter–gray matter signal difference for cortical gray matter of 47% ± 7%. The differential point spread function was narrowest for the spoiled gradient echo but slightly increased by 8% for vFA SSFP‐FID. For vFA SSFP‐FID, DLR offered a considerable decrease in the overall scan time from 5:17 min down to 2:46 min without noticeable image artifacts and degradations.

**Conclusions:**

At 0.55 T, a vFA MP‐RAGE variant using an SSFP‐FID kernel combined with a DLR method offers excellent prospects for rapid T_1_‐weighted whole brain imaging in less than 3 min with nearly 1 mm (1.12 × 1.17 × 1.25 mm^3^) isotropic resolution.

## 1 INTRODUCTION

MP‐RAGE MRI was introduced by Mugler and Brookeman in 1990^1^ as a novel volumetric technique featuring a MP step and a subsequent RAGE readout, as introduced by Haase et al. for snapshot FLASH MRI in 1989.^2^ As a volumetric acquisition, the MP‐RAGE repetitively imprints the desired tissue contrast onto the magnetization, followed by an image data acquisition during the approach to steady state, typically covering one partition of the k‐space volume. Optional delay periods (dead times) prior to and following the RAGE readout were also suggested for (partial) magnetization recovery. Generally, image encoding during a transient state will act like a k‐space filter, leading in the best‐case scenario only to broadening (blurring) of the point spread function (PSF) but in the worst case potentially degrading the image quality severely.^3^

Initially, MP‐RAGE was introduced as a 3D technique applicable throughout the body, and T_1_‐weighted imaging was demonstrated at 1.5 T for the abdomen and the brain.^1^ For the brain, the suggested MP‐RAGE setup featured an inversion preparation with an initial 500 ms delay, followed by a low‐flip angle spoiled gradient echo (SPGR) readout along the phase‐encoding direction and a 1 s recovery period at the end. The scan took 5:55 min for 1.41 × 1.95 × 0.98 mm^3^ resolution. Shortly after, Mugler and Brookeman started to investigate highly anisotropic MP‐RAGE scans to achieve scan times of approximately 1 min.^3^, ^4^ They noted that, depending on the delay time (TD), saturation rather than inversion pulses should be used—and that for centrally or short segmented reordering, high‐excitation angles could lead to higher SNR. Due to incomplete spoiling, however, the flip angle should be limited to about 10–15° for the SPGR readout. A possible solution to this problem could be coherent steady state free precession (SSFP) kernels,^4^ such as using the FID of SSFP (SSFP‐FID), also known as *FISP*.^5^ In follow‐up work,^3^ it was demonstrated that variable flip angle (vFA) SSFP‐FID kernels for rapid T_1_‐weighted MP‐RAGE MRI of the brain showed high potential not only in terms of SNR but also outperformed the white matter–gray matter signal difference (WGSD) of 3D SPGR imaging by about 41%. It was, however, also noted that T_1_‐weighted MP‐RAGE MRI of the brain with SSFP‐FID kernels exhibited some intensity artifact behavior that needed to be addressed.

In summary, considerable effort has been made to explore different design concepts for MP‐RAGE MRI. Due to the magnetization preparation step, imaging is predominantly performed in the transient rather than in the steady state, especially for the center of k‐space. Consequently, image contrast depends sensitively on internal parameters, such as relaxation times (and thus the field strength), as well as on the scan parameters themselves.^6^ For T_1_‐weighted MP‐RAGE MRI of the brain, the optimal preparation flip angle depends on the readout acquisition time and the TD, becoming lower than a simple inversion for short acquisition time and TD. A waiting time (TW) allows for additional contrast control, and the signal behavior during the RAGE readout can be manipulated; vFA SSFP‐FID kernels, for instance, may yield improved SNR and WGSD compared to SPGR‐kernels.^7^

Nowadays, at clinical field strength of 1.5–3 T, MP‐RAGE is one of the most commonly used sequences for T_1_‐weighted MRI of the brain, typically using magnetization inversion in conjunction with a train of low‐flip angle SPGR readouts, as initially proposed.^1^ Only recently, however, we have shown that SSFP‐FID kernels with constant flip angles show some benefit for T_1_‐weighted MP‐RAGE MRI of the brain at low field.^8^ In this study, building on seminal early work, we investigated the prospects of different coherent SSFP kernels in combination with a vFA strategy for improved SNR and WGSD MRI of the brain at 0.55 T. Moreover, for the best‐performing MP‐RAGE variant, the possibility of accelerated imaging in combination with a deep‐learning reconstruction (DLR) method was further evaluated.

## 2 METHODS

MRI was performed on a commercially available 0.55 T low‐field MR‐system (Magnetom Free.Max, Siemens Healthineers, Forchheim, Germany). A 12‐channel head coil was used for signal reception. Written informed consent was obtained from five healthy volunteers: three for exploring SSFP‐kernel variants and two for investigating the prospects of deep learning. The study was approved by our local ethics committee.

### 2.1 MP‐RAGE sequence

**FIGURE 1:**
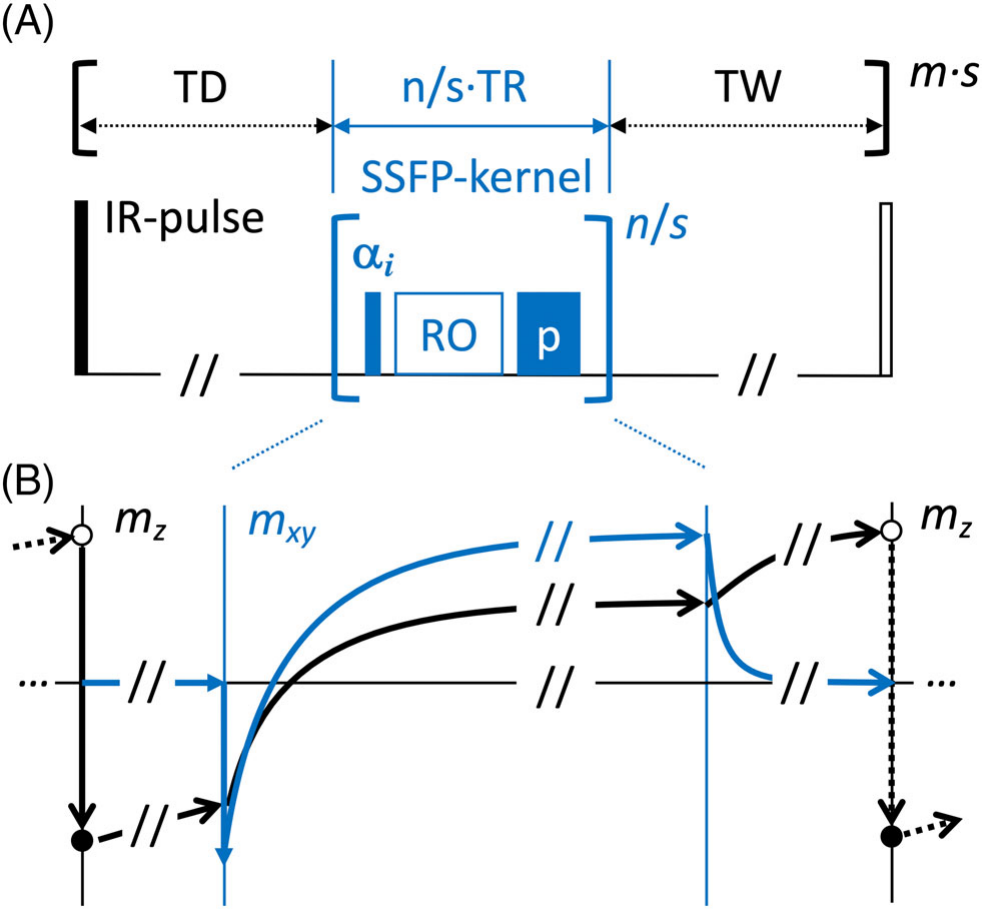
(A) Schematics of the MP‐RAGE sequence with different SSFP kernels used in this work. For bSSFP, *p* is zero, whereas for the FID and SPGR kernels it is non‐zero. A possibly segmented train of *n*/*s* kernels (with constant or varying flip angle, *α*_*i*_) is acquired along the inner encoding direction. The MP consists of a nonselective RF inversion pulse (including crusher gradients, not shown) and an optional TD. The RAGE readout is terminated by an optional TW (including crusher gradients, not shown). The entire MP‐RAGE unit is repeated *m·s* times along the outer encoding direction. (B) Sketch of a hypothetical time evolution of the magnetization within each MP‐RAGE unit: After each inversion, the magnetization qualitatively evolves along an IR‐curve, but note that the exact time evolution will depend on many parameters, such as relaxation and flip angles, including choice of the SSFP kernel (longitudinal magnetization is shown in black, transverse magnetization in blue). bSSFP, balanced SSFP; IR, inversion recovery; *m*, total number of outer encoding steps; MP, magnetization preparation; *n*, total number of inner encoding steps; *p*, crusher gradient moment; *s*, number of segmentations; SPGR, spoiled gradient echo; SSFP, steady state free precession; TD, delay time; TW, waiting time.

A generic diagram of the 3D MP‐RAGE sequence with different SSFP kernels is shown in Figure 1A. After a nonselective, adiabatic, inversion pulse and an optional TD, an SSFP‐RAGE readout is repeated *n*/*s* times along the inner encoding direction (i.e., either sequentially along the phase [phase‐encoding] or the partition [3D] direction), where *n* and *m* denote the number of inner and outer phase encoding steps and *s* indicates the number of segments. The SSFP kernel has a fixed TR and either constant (*α*) or vFAs (*α*_*i*_). For SPGR, a linear RF phase increment of 50° is used in combination with a crusher gradient moment *p* (2*π* within each voxel) after the readout. For the SSFP‐FID, the RF phase increment is set to zero. For the balanced SSFP (bSSFP) kernel, a constant RF phase increment of 180° is used (alternating phase bSSFP) and the crusher gradient moment *p* is set to zero. An optional TW completes the repetitive MP‐RAGE preparation and readout period that is repeated *m·s* times along the outer encoding direction.

A qualitative sketch of the time course of the magnetization is shown in Figure 1B. After the inversion pulse, crusher gradients are used to null the transverse magnetization. During the TD interval, the longitudinal magnetization starts to recover before the RAGE readout drives the longitudinal and transverse magnetization along an SSFP‐characteristic recovery toward the steady state (note that, e.g., depending on the flip angle and kernel used, this recovery is not necessarily a simple exponential). For bSSFP only, a linear ramp down of 20 dummy TR is used to stabilize and refocus the magnetization onto the longitudinal direction. Generally, the RAGE readout is terminated by crusher gradients and an optional recovery period (TW).

As investigated in prior work at 0.55 T, the TD and TW times were set to zero^8^; the SSFP‐FID was segmented by a factor of two; and an effective TI is reported that refers to the time between the inversion and the encoding of the center of k‐space along the inner direction.

### 2.2 Custom MP‐RAGE with SPGR‐kernel for technique comparison

As a reference, an optimized custom MP‐RAGE sequence was used, featuring an SPGR‐kernel with constant flip angle as described above. Compared to the standard product MP‐RAGE, this custom MP‐RAGE sequence featured a lower bandwidth and optimized gradient switching times, yielding an approximate 25% gain in the SNR and a 15% increase in the WGSD.^8^

### 2.3 Custom MP‐RAGE coherent SSFP kernel variants

For MP‐RAGE MRI with coherent SSFP kernels, either the FID or bSSFP was used. In related work, it was already shown that the MP‐RAGE with a constant flip angle SSFP‐FID kernel offered a little bit less SNR (−9%) but increased WGSD (+22%) as compared to the SPGR variant.^8^ For the vFA SSFP‐FID and bSSFP kernels, the flip angle of the RAGE readout was linearly incremented (along the inner encoding direction) from 1° to a final excitation angle (*α*), reached at the k‐space center, and then kept constant.

### 2.4 Imaging Protocols

Detailed MP‐RAGE protocol settings are given in Table S1.

### 2.5 Deep Learning Reconstruction (DLR)

For the image generation from undersampled k‐space data, a deep learning–based architecture inspired by variational networks^9^ was employed, as described in detail in the work of Fujita et al.^10^ The algorithm performs six iterations that alternate between parallel imaging–based data consistency and neural network–based image enhancement (including local denoising) by evaluating the recursive relation

(1) $$x_{n+\frac{1}{2}}=\underset{x}{\text{argmin}}\left({\left\|\mathrm{Ax}-y\right\|}^{2}+\frac{1}{\lambda_{n}^{2}}{\left\|x-x_{n}\right\|}^{2}\right)$$

(2) $$x_{n+1}=U^{\left(n\right)}\left(x_{n+\frac{1}{2}}\right),$$

starting from $x_{0}=0$. Here, A is the system operator that relates the image volume x to the acquired k‐space data y using precalculated coil sensitivity maps, $\lambda_{n}$ are step sizes that enforce similarity to the previous image volume $x_{n}$, and $U^{\left(n\right)}$ are 3D U‐nets.^11^ The overall architecture was trained end‐to‐end in a supervised manner on a dedicated GPU server with L1‐norm as loss function and Adam as optimizer using about 1000 fully sampled datasets obtained from healthy volunteers in different body regions utilizing routine 1.5 and 3 T scanners (Magnetom scanners, Siemens Healthineers). The obtained model parameters were then exported in ONNX format and integrated into a research application provided by Siemens Healthineers for prospective use on the scanner.

### 2.6 Differential point spread function (dPSF)

The differential point spread function refers to a hypothetical situation where one pixel of a substance B is embedded in the middle of a line of pixels with substance A.^12^ Edge smearing is caused by a nonconstant time difference evolution of the magnetization between the substances A (*s*_*A*_(*k*)) and B (*s*_*B*_(*k*)) during the RAGE readout, effectively acting as a k‐space filter. Assuming similar proton densities for both substances, the dPSF can be approximated by the discrete Fourier transform (DFT) of the corresponding differences in the time evolutions *DFT*(*s*_*B*_(*k*)–*s*_*A*_(*k*)).

### 2.7 Simulations

Ideal inversion and ideal spoiling of transverse magnetization were presumed after TD and after TW. The time course of the magnetization during the RAGE readout was simulated for both SPGR and SSFP‐FID using an extended phase graph formalism,^13^, ^14^ whereas for bSSFP 3 × 3 rotation and relaxation matrices from the piece‐wise constant integrated Bloch equation were used.^15^ A few MP‐RAGE trains (typically about four) were simulated in order to reach steady state conditions prior to each IR pulse. For white matter, PD/T_1_/T_2_ of 0.72/500 ms/90 ms were assumed; and for gray matter, PD/T_1_/T_2_ of 0.82/800 ms/110 ms were assumed.^16^, ^17^ Other simulation parameters are listed in the Table S1.

### 2.8 SNR and WGSD assessments

For all SSFP kernels except bSSFP, the same timing parameters, scan duration, and bandwidth were used (see Figure 1 and Table S1). As a result, the observed signal directly relates to the SNR. For bSSFP, the total readout time for the same encoding matrix was shortened due to the increase of the bandwidth ($\sqrt{93\;\left[\mathrm{Hz}\right]/298\;\left[\mathrm{Hz}\right]}$), but the scan time was subsequently increased using oversampling ($\sqrt{256/154}$). Taking into account the shorter scan duration ($\sqrt{318\;\left[s\right]/300\;\left[s\right]}$), this overall leads to an SNR penalty of 0.7416 for the MP‐RAGE with vFA bSSFP as compared to the other variants, and its signal has been accordingly rescaled prior to any visualization and calculation.

Signal differences between white and gray matter were estimated locally within manually drawn regions of interest in frontal white matter and the caudate nucleus head, as well as for average white to average cortical gray matter based on automatic segmentation using FreeSurfer v2.2.0.^18^

## 3 RESULTS

Numerical simulations indicated that flip angles should be increased for the vFA coherent SSFP kernels as compared to the common SPGR variant. For the vFA SSFP‐FID variant, simulated WGSD peaked around 30°, whereas for bSSFP the optimal flip angle was found to be around 40°.

**FIGURE 2:**
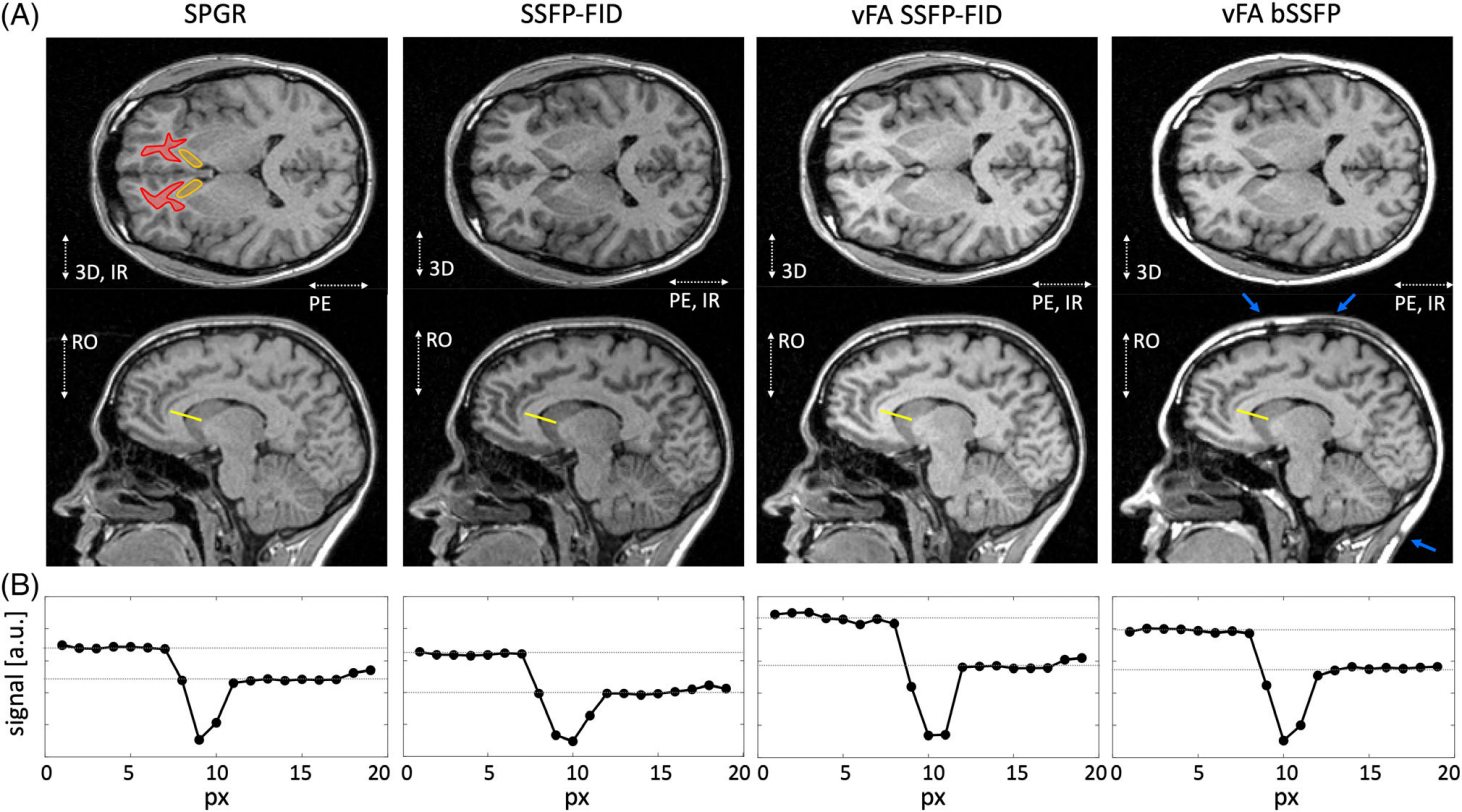
(A) Example axial and sagittal MP‐RAGE images using either an SPGR kernel, an SSFP‐FID kernel with constant flip angle, an SSFP‐FID kernel with vFA SSFP‐FID, or a vFA bSSFP. For protocol details, see Table S1. The windowing was adjusted to directly reflect the white matter SNR. The orange (caudate nucleus head) and red (frontal white matter) ROIs indicated in the axial SPGR variant are used for local SNR and local contrast‐to‐noise estimation. The blue arrows in the vFA bSSFP kernel variant indicate banding artifacts in fatty tissue. The individual encoding directions (RO, PE, partition encoding: 3D) along the IR are marked in the images. (B) MP‐RAGE line profiles (see yellow line) are shown for transitions from the corpus callosum to the CSF and to the caudate nucleus head (gray matter). PE, phase encoding; RO, readout; ROI, region of interest; vFA, variable flip angle.

Axial and sagittal example T_1_‐weighted MP‐RAGE brain images are shown for all RAGE variants in Figure 2A, together with signal profiles illustrating the transition from white matter (corpus callosum) to CSF and gray matter (caudate nucleus head) in Figure 2B. The profiles support the visual impressions that the vFA SSFP‐FID yields the highest signal (and thus SNR) and WGSD. Averaged across three volunteers (mean ± std), vFA SSFP‐FID showed a local relative SNR improvement in frontal white matter of 29% ± 3% and a relative increase in the signal difference between frontal white matter and the caudate nucleus head of 65% ± 15%, compared to the reference SPGR variant (detailed in Table S2). The local SNR and WGSD improvements are further corroborated by the same three volunteers with the results using an automated segmentation. For the whole brain, the SNR for white matter is increased by 21% ± 2%, and the signal difference to the cortical gray matter is improved by 47% ± 7%. A summary of the whole brain relative SNR and relative WGSD is given in Table 1 for all MP‐RAGE variants.

**TABLE 1:** Average SNR values for brain WM and cortical GM, and average relative WGSD values between WM and cortical GM for all kernel variants.

Kernel	WM SNR^a^	GM SNR^a^	WGSD^b^
SPGR	1.00(0) ± 0.02	0.76(4) ± 0.02	1.00(0) ± 0.10
SSFP‐FID	0.90(3) ± 0.02	0.62(3) ± 0.02	1.18(9) ± 0.11
vFA SSFP‐FID	1.21(2) ± 0.02	0.86(4) ± 0.01	1.47(3) ± 0.07
vFA bSSFP	1.12(0) ± 0.02	0.80(4) ± 0.01	1.33(5) ± 0.07

Abbreviations: bSSFP, balanced SSFP; GM, gray matter; SPGR, spoiled gradient echo; SSFP, steady state free precession; vFA, variable flip angle; WGSD, white matter–gray matter signal difference; WM, white matter.

^a^ Relative to SPGR brain white matter SNR.

^b^ Relative to SPGR WGSD for brain white matter–cortical gray matter; (mean ± std averaged over three volunteers).

**FIGURE 3:**
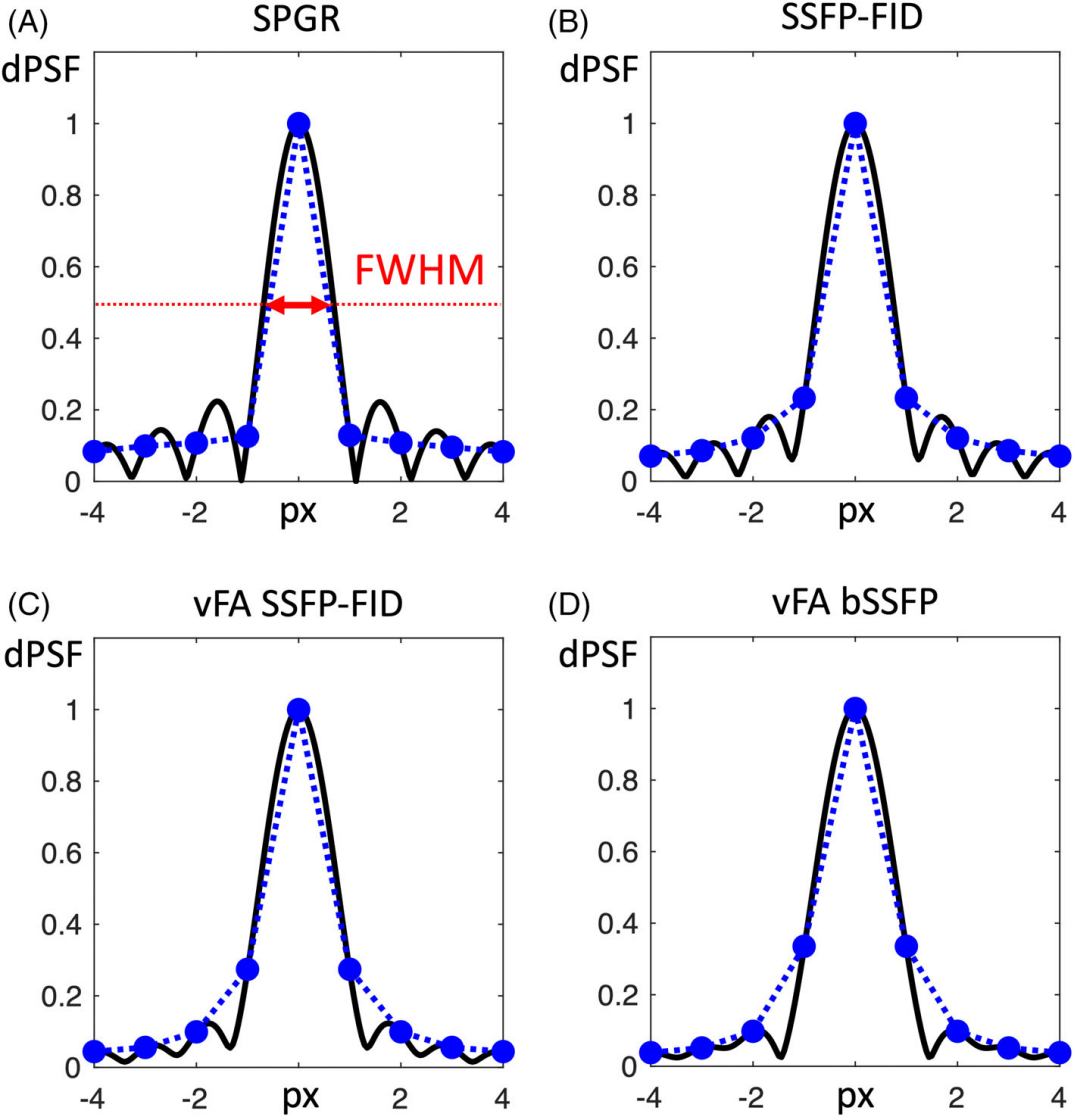
dPSF for data sampling along the IR curve for all four MP‐RAGE variants (blue points show the simulated discrete dPSF, and the black represents the interpolated dPSF using zero‐filling in k‐space prior to Fourier transformation). For the interpolated dPSF, FWHM was: (A) 1.38 px for the SPGR‐kernel, (B) 1.49 px for the SSFP‐FID kernel, (C) 1.49 px for the vFA SSFP‐FID kernel, and (D) 1.60 px for the vFA balanced SSFP (bSSFP) kernel. dPSF, differential point spread function; px, pixel.

The simulated dPSF for all investigated MP‐RAGE variants are shown in Figure 3. For the presumed relaxation times, the SPGR variant yielded the narrowest dPSF with a FWHM of 1.38 pixels (px). For both SSFP‐FID variants (either with constant or with vFA), the dPSF increases slightly by about 8% to 1.49 px and by about 16% to 1.60 px for the variant with the bSSFP kernel. For vFA SSFP‐FID as compared to the SPGR, the increase in the line broadening is considerably lower than the overall gain in the SNR for white matter.

**FIGURE 4:**
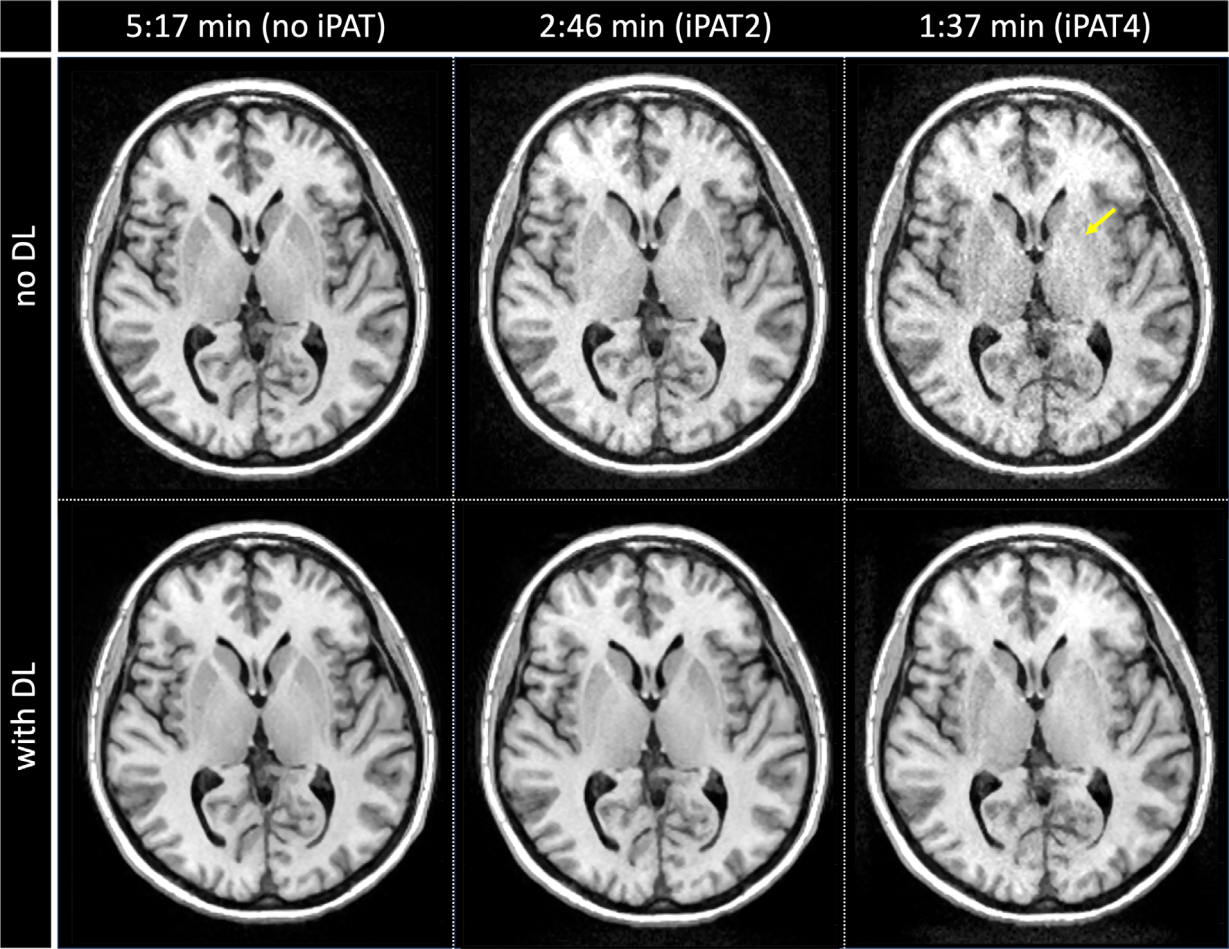
Assessment of deep learning–based denoising on MP‐RAGE using a vFA SSFP‐FID kernel. Illustrative axial example images are shown. (A) No acceleration. (B) iPAT2. (C) iPAT4. iPAT, integrated parallel acquisition technique. (In the Figure S3 corresponding example, sagittal images are shown). The prospects of high acceleration factors (iPAT4) appear limited by the loss of the contrast in the basal ganglia (yellow arrow)

Overall, the MP‐RAGE with the vFA SSFP‐FID kernel offered the highest SNR and WGSD. Therefore, its potential for acceleration using twofold (*R* = 2) or fourfold (*R* = 4) undersampling combined with a DLR was further investigated. Example axial images are shown in Figure 4, and example sagittal images are collected in the Figure S1. Overall, no noticeable, visually apparent image degradation is observed for the *R* = 2 acceleration in combination with the proposed DLR method. However, the *R* = 4 scan showed some minor image degradation and noise amplification, especially in the basal ganglia.

## 4 DISCUSSION

A general characteristic of the MP‐RAGE sequence is the vast parameter space, which complicates the search for an optimal design. In early work concentrating on the optimization of T_1_‐weighted MP‐RAGE MRI of the brain, different SSFP‐kernels in combination with vFA schemes were exploited. It was observed that echo shaping with vFA coherent gradient echoes offered higher SNR and WGSD than constant flip angle incoherent ones, but coherent MP‐RAGE was also more sensitive to gradient imperfections, such as eddy currents, or RF field inhomogeneities.^3^ This, in combination with sufficient SNR at high field, has possibly led to the situation that T_1_‐weighted MP‐RAGE MRI of the brain has become established using a simple train of constant and low‐flip angle SPGRs at high magnetic fields.

Recently, lower field scanners (<1 T) have regained increased interest and have freshly emerged into the market. MRI at these lower field strengths is inherently challenged by reduced SNR compared to high‐field MRI. This can be counteracted by increasing the scan time, decreasing the resolution or by a dedicated sequence design to partially compensate for the loss in SNR, as performed in this work. Moreover, prominent B_1_ field inhomogeneities are to a large part mitigated. As a result, it can be expected that the reported substantial signal intensity variations for T_1_‐weighted MP‐RAGE at high‐field strength (3 T and above) are no longer a concern, possibly rendering bias‐field correction such as using a self‐bias field‐corrected sequence^19^ (called *MP2RAGE*, which has a substantially longer acquisition time than MP‐RAGE) unnecessary.

In this work, a rather pragmatic strategy was explored for the vFA scheme using a simple linear ramp up to the center of k‐space and then keeping the flip angle constant. This offered the major advantage that the parameter space for optimization is not increased. Despite its simplicity, this method clearly demonstrated the potential of vFAs to increase the WGSD in MP‐RAGE sequences. Obviously, a dedicated search for WGSD‐optimized flip angle modulations, similar to methods previously explored at high fields,^3^, ^7^ would likely yield even greater improvements.

In addition to the optimization of the acquisition strategy alone, the prospects of a conservative DLR of undersampled k‐space data were explored for the best performant MP‐RAGE variant. In healthy volunteers, twofold acceleration with DLR yielded images without major image degradation, whereas some artifacts became apparent with fourfold acceleration (*R* = 4). Overall, the suggested twofold accelerated MP‐RAGE protocol using a vFA SSFP‐FID kernel in combination with the proposed DLR method appears very appealing for clinical evaluation.

## 5 CONCLUSION

Using a segmented RAGE readout with a vFA SSFP‐FID kernel for T_1_‐weighted MP‐RAGE MRI of the brain outperforms the common MP‐RAGE design at 0.55 T, both in terms of SNR and WGSD. Moreover, combining this sequence with DLR enables whole‐brain imaging with approximately 1 mm isotropic resolution (1.12 × 1.17 × 1.25 mm^3^) in under 3 min.

## Supporting information


**Table S1.** Scan parameters for magnetization prepared rapid gradient‐echo (MP‐RAGE) variants. All scanning was performed in sagittal orientation and the same non‐selective (hard) RF pulse was used for inversion with all steady state free precession (SSFP)‐kernel variants. No delay times were used before or after the inversion RF pulse. “TI_eff_” indicates the inversion time (the time between the inversion pulse and the sampling of the k‐space center), “PE” the direction of phase encoding and “3D” the direction of partition encoding.
**Table S2.** Local relative signal‐to‐noise ratio (SNR) estimates for frontal white matter and its relative white matter‐gray matter signal difference (WGSD) to deep gray matter (caudate nucleus CN head) for all kernel variants (for definition of regions of interest (ROIs), see Figure 2A).
**Figure S1.** Assessment of deep learning based denoising on magnetization prepared rapid gradient‐echo (MP‐RAGE) using a variable flip angle steady state free precession (SSFP)‐FID (vFA SSFP‐FID) kernel. Illustrative sagittal example images are shown. (A) No acceleration. (B) iPAT2. (C) iPAT4.
