# Supplementary material for: Optimized T1 ‐weighted MP‐RAGE MRI of the brain at 0.55 T using variable flip angle coherent gradient echo imaging and deep learning reconstruction
Source: Magn Reson Med. 2025 Sep 29;95(2):1169–76. doi: 10.1002/mrm.70109 (PMC12681286; doi:10.1002/mrm.70109)
Supplement: Supplementary file 1 — Table S1. Scan parameters for magnetization prepared rapid gradient‐echo (MP‐RAGE) variants. All scanning was performed in sagittal orientation and the same non‐selective (hard) RF pulse was used for inversion with all steady state free precession (SSFP)‐kernel variants. No delay times were used before or after the inversion RF pulse. “TIeff” indicates the inversion time (the time between the inversion pulse and the sampling of the k‐space center), “PE” the direction of phase encoding and “3D” the direction of partition encoding. Table S2. Local relative signal‐to‐noise ratio (SNR) estimates for frontal white matter and its relative white matter‐gray matter signal difference (WGSD) to deep gray matter (caudate nucleus CN head) for all kernel variants (for definition of regions of interest (ROIs), see Figure 2A). Figure S1. Assessment of deep learning based denoising on magnetization prepared rapid gradient‐echo (MP‐RAGE) using a variable flip angle steady state free precession (SSFP)‐FID (vFA SSFP‐FID) kernel. Illustrative sagittal example images are shown. (A) No acceleration. (B) iPAT2. (C) iPAT4. [file MRM-95-1169-s001.docx]

**Table S1**: Scan parameters for MP-RAGE variants. All scanning was performed in sagittal orientation and the same non-selective (hard) RF pulse was used for inversion with all SSFP-kernel variants. No delay times were used before or after the inversion RF pulse. “TI_eff_” indicates the inversion time (the time between the inversion pulse and the sampling of the k-space center), “PE” the direction of phase encoding and “3D” the direction of partition encoding.

| **SSFP-Kernel** | **SPGR** | **SSFP-FID** | | | | **bSSFP**** |
| --- | --- | --- | --- | --- | --- | --- |
| FOV [mm^3^] | 250 × 250 × 176 | | | | | |
| Acq. resolution [mm^3^] | 1.12 × 1.17 × 1.25 | | | | | |
| Slice oversampling [%] | 9.1 | | | | | 81.8*** |
| Matrix size | 224 × 212 × 154 | | | | | 224 × 212 × 256 |
| TR [ms] | 10.80 | | | | | 5.40 |
| Bandwidth [Hz/px] | 93 | | | | | 298 |
| IR encoding direction | 3D | PE | | | | |
| TI_eff_ [ms] | 832 | 572 | | | | |
| Segments | 1 | 2 | | | | 1 |
| Partial Fourier (Direction) | 7/8 (PE) | 7/8 (3D) | | | - | 7/8 (3D) |
| Variable flip angle (vFA) | - | - | yes | yes | | yes |
| Flip angle | 20 | 20 | 30 | 30 | | 40 |
| RF pulse duration [us] | 200 | 200 | 300 | 300 | | 400 |
| iPAT* | - | - | | 2 (PE) | 2 x 2 | - |
| DLR | - | - | | yes | | - |
| Acquisition time [min] | 5:18 | 5:17 | | 2:46 | 1:37 | 5:00 |

*Using a separate 0:06 [min] SPGR reference scan for estimation of the coil sensitivity: 3D SPGR kernel with 24 x 24 central lines; TR = 10.8 ms, BW = 93 Hz/px; flip angle = 5°. The acquisition time includes the duration of the reference scan. The standard iPAT (GRAPPA) undersampling pattern was used for DLR. **For the bSSFP kernel, a ramp down of 20 dummy repetition times was played out for refocusing purposes at the end of the IR period together with a short crusher gradient. ***Oversampling adjusted to achieve similar scan times as with the other kernels.

**Table S2:** Local relative SNR estimates for frontal white matter and its relative WGSD to deep gray matter (caudate nucleus CN head) for all kernel variants (for definition of ROIs, see Figure 2A).

| Kernel | Frontal WM SNR* | CN Head SNR* | Local WGSD** |
| --- | --- | --- | --- |
| SPGR | 1.00(0) ± 0.02 | 0.76(8) ± 0.02 | 1.00(0) ± 0.08 |
| SSFP-FID | 0.96(6) ± 0.03 | 0.64(4) ± 0.02 | 1.38(8) ± 0.14 |
| vFA SSFP-FID | 1.29(4) ± 0.03 | 0.91(0) ± 0.03 | 1.65(6) ± 0.15 |
| vFA bSSFP | 1.20(5) ± 0.03 | 0.85(5) ± 0.02 | 1.50(9) ± 0.13 |

*Relative to SPGR SNR; **relative to SPGR WGSD (mean ± std averaged for three volunteers).

**
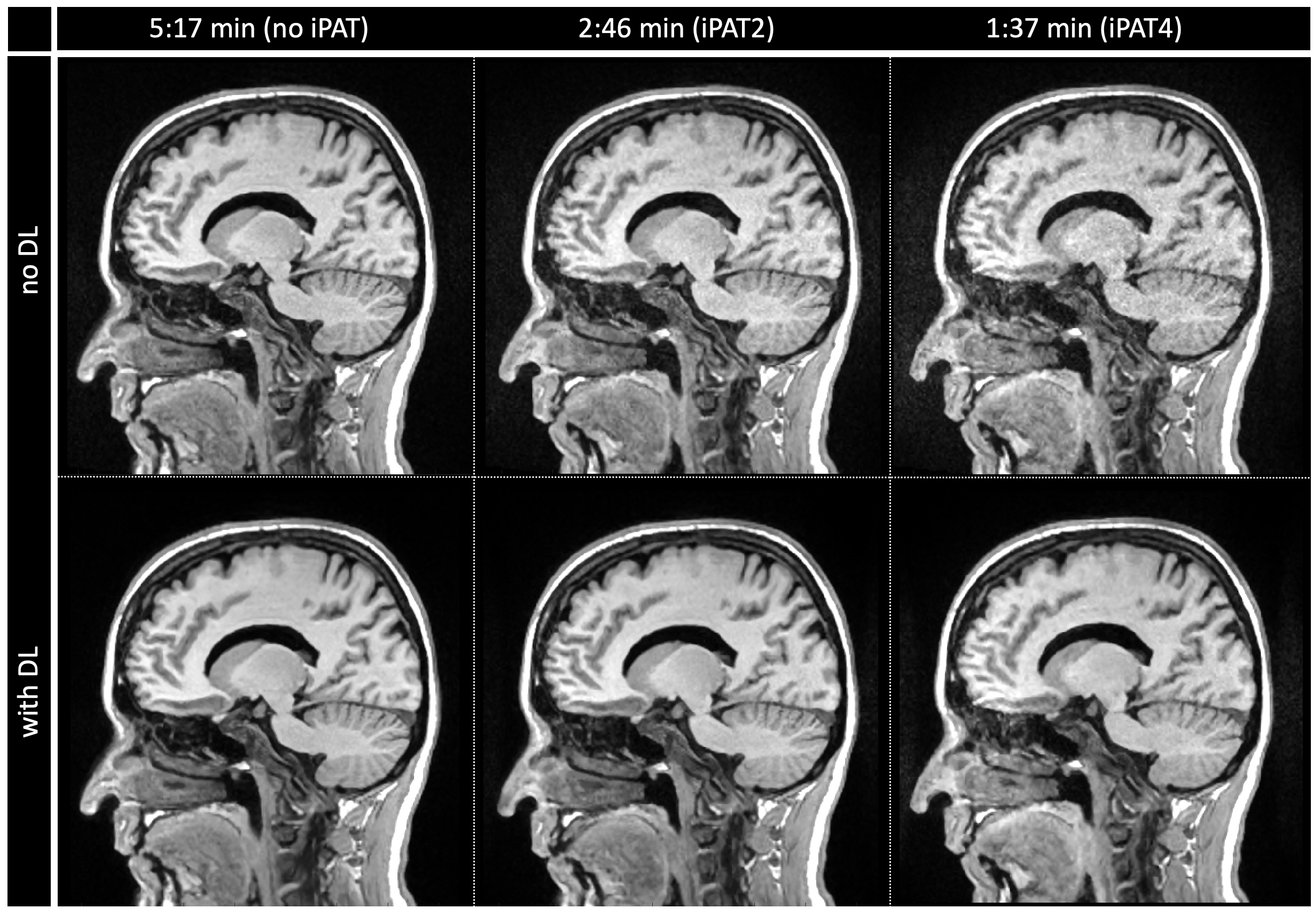
**

**Figure S1:** Assessment of deep learning based denoising on MP-RAGE using a variable flip angle SSFP-FID kernel. Illustrative axial example images are shown. A) No acceleration. B) iPAT2. C) iPAT4.
